# Hydrogen-plasma-induced Rapid, Low-Temperature Crystallization of μm-thick a-Si:H Films

**DOI:** 10.1038/srep32716

**Published:** 2016-09-07

**Authors:** H. P. Zhou, M. Xu, S. Xu, L. L. Liu, C. X. Liu, L. C. Kwek, L. X. Xu

**Affiliations:** 1 School of Energy Science and Engineering, University of Electronic Science and Technology of China, 2006 Xiyuan Ave, West High-Tech Zone, Chengdu, 611731, China; 2 Plasma Sources and Application Center, NIE, and Institute of Advanced Studies, Nanyang Technological University, 637616, Singapore; 3 Key Laboratory of Information Materials of Sichuan Province & School of Electrical and Information Engineering, Southwest University for Nationalities, Chengdu 610041, China; 4 Institute of Microstructure and Properties of Advanced Materials, Beijing University of Technology, Beijing, 100124, China; 5 Centre for Quantum Technologies, National University of Singapore, 119077, Singapore

## Abstract

Being a low-cost, mass-production-compatible route to attain crystalline silicon, post-deposition crystallization of amorphous silicon has received intensive research interest. Here we report a low-temperature (300 °C), rapid (crystallization rate of ~17 nm/min) means of a-Si:H crystallization based on high-density hydrogen plasma. A model integrating the three processes of hydrogen insertion, etching, and diffusion, which jointly determined the hydrogenation depth of the excess hydrogen into the treated micrometer thick a-Si:H, is proposed to elucidate the hydrogenation depth evolution and the crystallization mechanism. The effective temperature deduced from the hydrogen diffusion coefficient is far beyond the substrate temperature of 300 °C, which implies additional driving forces for crystallization, i.e., the chemical annealing/plasma heating and the high plasma sheath electric field. The features of LFICP (low-frequency inductively coupled plasma) and LFICP-grown a-Si:H are also briefly discussed to reveal the underlying mechanism of rapid crystallization at low temperatures.

High growth rate and low-temperature (less than 600 °C) fabrication of micro-/nanocrystalline (or polycrystalline) silicon has attracted worldwide fundamental and technological interest due to its broad applications in the fields of solar cells, thin-films transistors, image sensors etc^1^,^2^. Currently practical plasma deposition of microcrystalline silicon is technologically and commercially hindered by the difficulties in attaining high-growth-rate preparation of large-area films at low temperature^3^. Solid-phase crystallization (SPC)^4^,^5^,^6^,^7^ of amorphous hydrogenated silicon (a-Si:H) is one of the important alternatives to acquire high-quality polycrystalline silicon. There have been many reports on the SPC-fabricated polycrystalline silicon already utilized in thin film transistor^8^,^9^ and thin film solar cells^10^,^11^. However, the commonly used SPC conducted in a furnace requires a high temperature (>600 °C) and a long-time (>10 h) thermal annealing because the formation of crystalline nuclei in the precursor a-Si matrix has to overcome a large barrier energy of ~5 eV^12^,^13^. Thus, metal-induced crystallization (MIC)^9^,^14^, field-enhanced crystallization (FEC)^15^,^16^,^17^ and plasma-induced crystallization (PIC)^6^,^18^ have been proposed, and their feasibilities have been evidenced by the effective lowering of the crystallization temperature and the reducing of the incubation time for the formation of crystalline nuclei. Compared with MIC and FEC, PIC is more convenient, requiring only the introduction of hydrogen plasma instead of a catalyst or an additional field. A growing number of attempts have been made in hydrogen plasma-induced post-deposition crystallization of a-Si:H thin films^4^,^6^,^18^. Hydrogen plasma treatment causes numerous complicated reactions such as hydrogen diffusion/insertion and annihilation of strained Si-Si bonds^4^,^19^. An increase in the crystalline fraction by 10~15% was achieved by electron cyclotron resonance (ECR) hydrogen plasma treatment of ECR-CVD grown a-Si:H for 60 minutes at 325 °C^20^. The thermal crystallization time of a-Si:H is reduced by a factor of five by room temperature hydrogen plasma exposure^21^. Still, rapid and low-temperature crystallization of a-Si:H films is highly expected to obtain micro-/nanocrystalline silicon thin films to meet the needs in the field of large area electronics.

On the other hand, to date, the reported PIC of a-Si:H have been limited in very thin films (a few tens nanometer), where the in-diffusion of hydrogen was largely neglected^22^,^23^. However, in a-Si:H thin film solar cells, the underneath a-Si:H (at the level of micrometers) is exposed to hydrogen containing plasma during the subsequent deposition process, which inevitably influences the microstructures and properties of the underlying a-Si:H layers and overall cell efficiency^24^. In the case of a very thin a-Si:H film, researchers have reached a consensus that the competition between hydrogen insertion into the amorphous network and silicon etching by hydrogen exposure results in the occurrence of a hydrogen-rich surface layer with an ultimately steady thickness. With regard to micrometer-thick a-Si:H, in-diffusion of hydrogen into the deep region and associated effects on the microstructures and properties of the influenced layers are still expected. From the application prospective, a new PIC strategy for effective crystallization of micrometer thick a-Si:H is imperative.

In the present work, we report on controllable, rapid crystallization of hydrogenated amorphous silicon, directly induced by high-density inductively coupled plasma (ICP) of H_2_ at low temperatures. High-density hydrogen plasma generated by our in-house developed LFICP equipment, which features a high-density (up to ∼10^13^/cm^3^) plasma at low pressures^25^,^26^, and independent control of electron density and the energy of ions, is utilized to induce the crystallization of micrometer thick a-Si:H films by plasma exposure. The phase change and hydrogenation depth evolution with respect to treatment duration were studied. We observed a rapid and complete crystallization of a-Si:H at a low temperature of 300 °C. The hydrogen-associated kinetic mechanism in this crystallization process is outlined accordingly. The present work will be greatly beneficial to the fundamental and technological research of solid phase crystallization of a-Si:H.

## Results and Discussions

**Figure 1:**
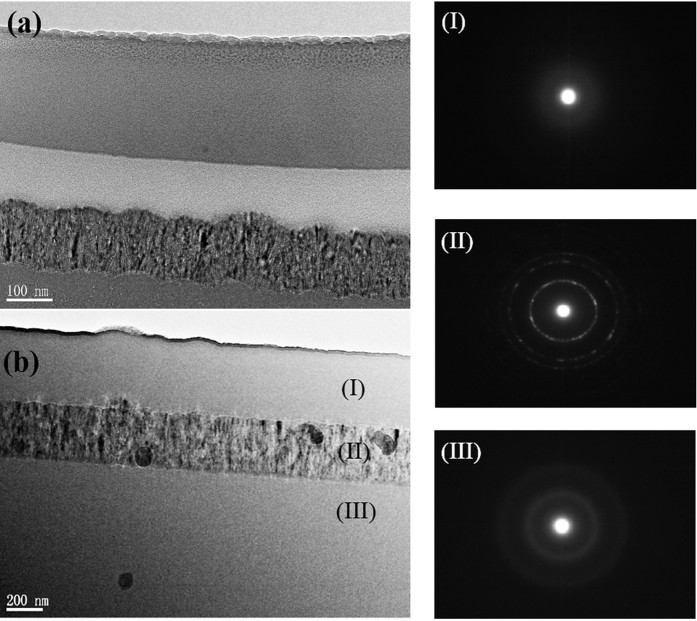
Cross-sectional TEM images of the 7-minute (**a**) and 15-minute (**b**) plasma treated a-Si:H films. The transmission electron diffraction pattern of the individual layers in multilayered structure (**b**) of a-Si/SiO_x_ (I)/polycrystalline Si (II)/a-Si (III) is represented in the right part of the figure.

The impact of hydrogen plasma exposure on the microstructure of a-Si:H films was observed by cross-sectional TEM measurements. Figure 1 shows the images of samples treated for 7 minutes (a) and 15 minutes (b). It is interesting that the cross-sections of the treated samples display evident multilayered structures. Comparing these two samples, one can find that the 7-min and 15-min samples are identified as four- and three-layer structures, respectively. Indeed, the 3.5-min sample also consists of four layers, and the 30-min sample’s structure is similar to that of 15-min sample. That is, the final layered structure is strongly dependent on the treatment duration. The sandwiched structure of the 15-min sample was further characterized by transmission electron diffraction measurement, as shown in the right part of Fig. 1 (I, II, III correspond to the three different regions as denoted in Fig. 1(b) from top to bottom). There is no distinguished diffraction ring in the diffraction pattern of region (I), indicating the fully amorphous structure of the top layer (I). In contrast, region (II) shows a distinct diffraction rings, which implies that the middle layer (II) is highly crystallized by the plasma treatment. The diffraction pattern of region (III) shows two diffused diffraction rings, corresponding to an amorphous-dominant structure of the bottom layer (III). In order to analyze the elemental compositions of three layers, X-ray photoelectron spectroscopy (XPS) measurements were conducted to each layer. The survey scan patterns and the narrow-band scan patterns for the state of Si*2p* shown in Figure S2 (see the Supplementary Information) reveal that the top layer (I) contains a substantial composition of O and an insignificant composition of C besides the primary element Si, and layer (II) and (III) are chemically composed of the element Si only. In combination with the TEM results, the top layer (I) can be assigned as a-Si/SiO_x_. Actually, the crystal structure of the sandwiched layer shown in Fig. 1(a,b) can also be observed from the cross-sectional TEM images. The crystallized regions display evident columnar structure, which is frequently observed in PECVD-grown microcrystalline silicon films^27^. This finding seems to indicate that the present post-crystallization is similar to PECVD microcrystalline silicon growth, for which three models, namely the surface diffusion, etching, and chemical annealing models have been proposed to explain the crystallization process^28^. As such, the 2^nd^ layer in Fig. 1(a) is possibly an incubation (transition) layer^14^,^29^ between the top a-Si/SiO_x_ and the intermediate crystallized layer. Detailed transmission electron diffraction measures revealed its amorphous nature. A prolonged hydrogen plasma treatment (>15 minutes) completely eliminates this incubation layer, as shown in Fig. 1(b). However, the transition orientation to crystalline or complete amorphous phase needs further clarification.

In Fig. 1(a,b), the plasma-treated samples show a rough surface (~5 nm roughness). This is due to the etching of hydrogen on a-Si:H surface, and consistent with the previous reports in literature^22^. Detailed atomic force microscope (AFM) measurements (shown in the Supplementary Information) show that the root mean square (RMS) roughness kept comparatively stable when the treatment duration was more than 7 minutes.

**Figure 2:**
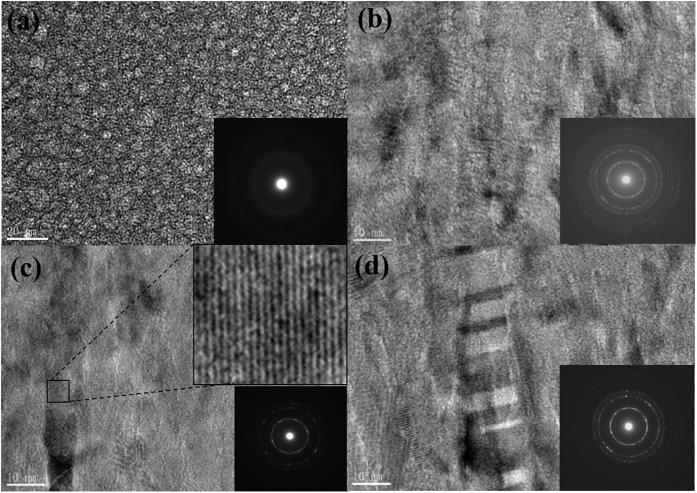
HRTEM images and corresponding electron diffraction patterns (inset) of the middle crystallized layer in (**a**)-Si:H films treated by hydrogen plasma for durations of 7 (**b**) 15 (**c**) and 30 minutes (**d**). The case of as-deposited a-Si:H (**a**) is also included for comparison. An enlarged image of a crystallized region is also inset in (**c**).

Figure 2 shows high-resolution (HR) TEM images of the crystallized layer in the samples treated for 7-min (b), 15-min (c) and 30-min (d), respectively. The case of as-deposited film is included in Fig. 2(a). The diffraction pattern in the inset of Fig. 2(a) demonstrates the amorphous structure of the as-deposited film, in which uniformly distributed amorphous silicon nanoparticles with size of ~3 nm are observed. The plasma treatment leads to the crystallization in the sandwiched layer, as demonstrated in Fig. 2(b,c), in which evident lattice fringes shows the crystalline attribution of the dark region. As an example, a crystallized region (marked by a small square in Fig. 2(c)) in the 15-min treated sample is magnified in the inset of Fig. 2(c). The inset of Fig. 2(b) represents a variety of diffraction rings, indicative of poly-crystalline structure without a preferred orientation in the 7-min sample. After a prolonged treatment up to 30 minutes, the 1^st^, 2^nd^, 3^rd^ rings (in an inside-outside order) corresponding to the (111), (220), and (311) orientations, respectively, become more and more prominent, indicating an increasing crystallinity of the crystallized layer. Comparing the inset of Fig. 2(d) with that of Fig. 2(c), one can see that the 1^st^ ring associated (111) orientation becomes the preferred growth orientation of the crystallites. This leads us to conclude that the plasma treatment duration is capable of controlling the preferred growth orientation, which is affected by the deposition parameters such as discharging power, hydrogen dilution ratio and reactive gas pressure in a conventional PECVD growth of microcrystalline silicon.

**Figure 3:**
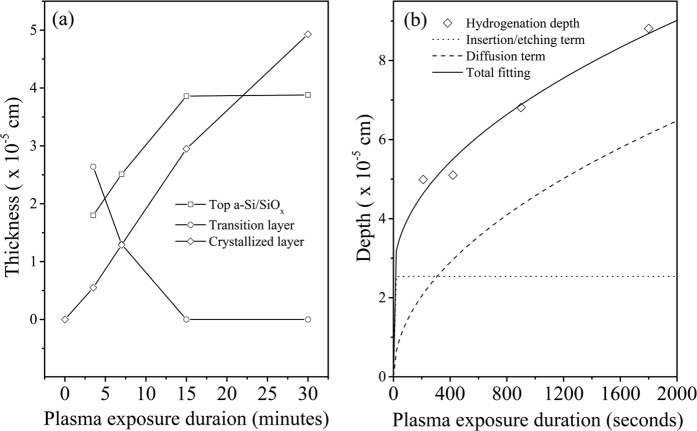
The time evolution of the thickness (**a**) of the individual layers, i.e. the top a-Si/SiO_x_, the middle transition, and bottom crystallized layer. (**b**) shows the hydrogen transport depth (open diamonds) into the sample under different plasma exposure durations, and the fitting (solid line) by the model including the contribution of hydrogen insertion/etching (dotted line) and diffusion (dashed line).

The thicknesses of the individual layers, including the top amorphous layer, intermediate incubation/transition layer, and the crystallized layer, in the treated sample were recorded from the cross-sectional TEM measurements and shown in Fig. 3(a). The top a-Si/SiOx layer gradually extends into the bulk in the range of 1.5–15 minutes, and saturates at the point of about 15 minutes. The thickness of the transition layer shows a converse tendency to that of the top a-Si/SiO_x_ layer. Meanwhile, the crystallized layer widens almost linearly with treatment duration with an approximate crystallization rate of ∼17 nm/minute. Obviously, the present method based on high-density hydrogen plasma enables rapid and highly controllable crystallization for a-Si:H.

As mentioned above, the multilayered structure of the hydrogen plasma-treated a-Si:H is similar to the case of PECVD-grown microcrystalline silicon, which includes four growth phases: incubation, nucleation, growth, and steady state^30^. The incubation (transition) layer is considered to be a remnant of the incubation phase, and many methods have been explored to eliminate it^31^. For example, layer-by-layer RF PECVD technique^32^ can achieve a fully crystallized interface through alternating sequences of a-Si:H deposition and hydrogen-plasma exposure. In our experiments, a short-time post-deposition hydrogen plasma introduces an incubation layer between the crystallized and the top amorphous layer. However, a continuous treatment makes the incubation layer fade out. The structure change in the underlying layers by plasma is a long-range effect of hydrogen that diffuses through the subsurface of the treated samples and penetrates into the deep region. As such, the top a-Si/SiO_x_ should concurrently be intensively influenced by the ion bombardment from the high-density hydrogen plasma generated by the present LFICP source. In contrast to our case, Kalache *et al*.^30^ observed an ion-induced amorphization effect in the subsurface of PECVD-grown microcrystalline silicon due to the ion bombardment effect. According to our TEM results shown in Figs 1 and 2, the hydrogen associated chemical annealing dominates over the ion-induced amorphization and results in the increased crystallized zone (widened crystallized layer). From the point view of hydrogen transport, the sum of the thickness of the top a-Si/SiO_x_ layer, transition layer and crystallized layer, which are influenced by hydrogen atoms, should mark the depth that hydrogen atoms penetrate into the bulk a-Si:H.

Let us consider anew the hydrogen kinetics in a-Si:H. Excess hydrogen atoms from the plasma can break strained Si-Si bonds with Si-H bond formation in a insertion process. This hydrogenated surface layer can further react with hydrogen atoms yielding ablation of SiH_4_ in a concurrent etching process^22^. The insertion and etching processes compete with each other during hydrogen plasma processing and reach a compromise with a self-saturation at a certain hydrogenation thickness. However, this configuration is not suitable for micrometer-level films because of neglecting the diffusion contribution. In our experiment, the hydrogen transported deep into bulk a-Si:H up to 900 nm depth within 30 minutes. As is known, the mechanism of hydrogen diffusion is quite complex and not fully understood, because it is correlated with doping level, free carriers, metastable/equilibrium defects, H content in the films, etc^33^,^34^. Given that the diffusion depth of hydrogen in a-Si:H is approximated by the term of 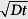 with diffusion coefficient *D* and diffusion time *t* (i.e. plasma treatment duration), the total transport depth of hydrogen can be expressed as the sum of the etching/insertion term^22^,^23^ and the diffusion term as follows,


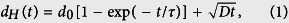


where *d*_*H*_ (*t*) is time dependent transport depth of hydrogen, *d*_*0*_ corresponds to the saturation depth in hydrogen insertion/etching process, and *τ* is a hydrogen insertion and etching related constant. This hydrogen kinetics relation is employed to model hydrogen transport in the present high-density plasma treatment experiment. The best fitting results are shown in Fig. 3(b) with the parameters of *d*_*0*_ = 254 nm, *τ* = 2.4 s and *D* = 2.1 × 10^−12^ cm^2^.s^−1^. The individual contributions from hydrogen insertion/etching and diffusion are included in Fig. 3(b) (dotted line and dashed line for insertion/etching and diffusion, respectively). As expected, the contribution of hydrogen insertion/etching saturates at the point of about 20 seconds (254 nm), and the diffusion contributes more to the transport depth, especially after the point of 5 minutes.

**Figure 4:**
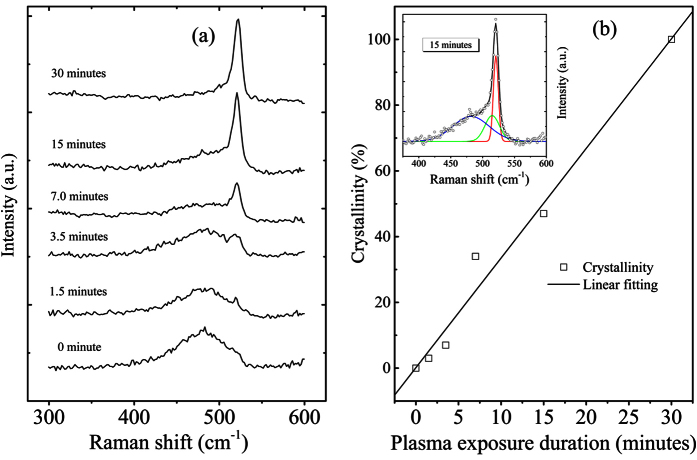
Micro-Raman scattering spectra of the samples plasma-exposed for different durations (**a**) and the exposure duration dependent average crystallinity (**b**) (denoted as open squares) estimated from the Raman scattering spectra shown in (**a**) as well as the linear fitting (solid line) of the average crystallinity.

The HRTEM and transmission electron diffraction patterns explicitly reveal the polycrystalline structure of the crystallized layer due to the plasma exposure. In order to quantitatively characterize the crystallization degree induced by hydrogen plasma, micro-Raman scattering measurements were performed on the as-deposited and plasma-treated samples deposited on both silicon and glass substrate. Figure. 4(a) shows the treatment duration dependent Raman scattering spectra of the films deposited on silicon films. As a matter of fact, the obtained spectra of the films deposited on silicon substrate and glass substrate coincide with each other, excluding the substrate effect on the crystallization process during the plasma processing.

The Raman scattering signal in the wavenumber region of 300–600 cm^−1^ comprises three components: the TO mode of amorphous silicon peak at about 480 cm^−1^, the intermediate phase around 510 cm^−1^, and the crystalline silicon phase near 521 cm^−1^. Accordingly, Raman spectra shown in Fig. 4(a) demonstrate a gradual phase transition from completely amorphous to highly crystal phase. However, we should keep in mind that the treated films are of a multilayered structure as shown above. Therefore, the Raman-spectra deduced crystallinity data are average values reflecting the average crystallization degree in the detection depth of the excitation wavelength. The crystallinity value was estimated by means of decomposing the Raman-spectra into three Gaussian shaped curves respectively corresponding to the three phases of silicon films. As an example, the decomposition of the 15-min samples is shown in inset of Fig. 4(b), where the blue, green and red curves denote the amorphous, intermediate and crystalline phase, respectively. The total fitting result (black curve) coincides well with the measured spectra. The crystallinity is estimated by the integrated areas ratio of crystalline phase plus intermediate phase to the total, and the as-calculated results are plotted in Fig. 4(b) (open squares) against treatment duration. Coinciding with the TEM experiments, the crystallinity is improved from 3% (as-deposited) up to 100% (30-min). A liner fitting is included in Fig. 4(b) giving an approximate crystallization ratio of ~0.033/minute.

It is worth noting that the present crystallization process occurs at a very low temperature of of 300 °C, being far below the crystallization temperature required (above 600 °C) for a-Si:H in a usual thermal annealing route. As shown above, a hydrogen kinetics for the multilayered structure formation was built and the extracted hydrogen coefficient is *D* = 2.1 × 10^−12^ cm^2^.s^−1^. It is well known that temperature is a key parameter in diffusion phenomena. The thermally activated hydrogen diffusion coefficient increases with temperature *T* (in K unit) in a way predicted by the Arrhenius law,


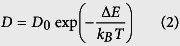


where *D*_0_ denotes the preexponential factor, *∆E* is the activation energy, and *k*_*B*_ is the Boltzmann constant. The value of the set (*D*_0_, *∆E*) has been systematically investigated, and the experimental results were summarized in Ref. 35. The set of *D*_0_ = 9.1 × 10^−3^ cm^2^.s^−1^, *∆E* = 1.49 eV for the hydrogen content *C*_*H*_ = 15.4% (approaching the hydrogen content estimated from Fourier transform infrared spectra in our as-deposited a-Si:H) is chosen to estimate the effective temperature *T*_*eff*_ in the present plasma treatment. The obtained value is *T*_*eff*_  = 779 K, which is 206 °C beyond the set temperature of the treated sample and the gas temperature (~200 °C, monitored through the top thermocouple as shown in the Supplementary Information, Figure S1). Actually, *T*_*eff*_ is insensitive to the value of (*D*_0_, *∆E*) in the broad range summarized in ref. 35. As a result, the deduced *T*_*eff*_ value is quite reliable. The difference between *T*_*eff*_ and the set temperature mainly originates from the chemical annealing of hydrogen plasma. In the present LFICP circumstances, high-density hydrogen can produce effective local plasma heating via the hydrogen recombination on the treated surface H + Si-H → H_2_ + Si_ with the dangling bond Si_. In addition, the sheath potential must also be partially responsible for this temperature difference. According to our previous plasma diagnostics^25^, the value of the electron number density *n*_*0*_ is very high (~9 × 10^12 ^cm^−3^), while the sheath potential (*u* ~ 13–17 V) is quite low in the present LFICP. The sheath thickness (*d*) in plasma is usually several Debye length *λ*_*DE*_, which is expressed as^36^


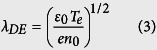


with *T*_*e*_, *ε*_*0*_, *n*_*0*_, and *e* being the plasma temperature, permittivity of vacuum, plasma density, and electron charge, respectively. As such, the equivalent electric field (*E* = *u/d*) applied onto our a-Si:H film is estimated to be ∼10^4 ^V/cm. Such high electric field is enough to accelerate the positive ions into the bulk a-Si:H and facilitate the hydrogen ion diffusion, and thus to enhance the chemical annealing and crystallization of the films. On the other hand, the high electric field may lead to a dielectric breakdown of the a-Si:H films and to associated Joule heat^15^. Consequently, the effective temperature *T*_*eff*_ may extremely exceed the set temperature. Compared with the aforementioned thermal annealing temperature above 600 °C required for crystallization, *T*_*eff*_ = 779 K (506 °C) is not high enough to trigger crystallization as rapid as that we observed. This can be explained in view of the features of the LFICP-grown a-Si:H precursor for plasma treatment. According to the FTIR spectra in the wavenumber range of 500–750 cm^−1^ (as shown in the Supplementary Information), the hydrogen content of the as-deposited a-Si:H film is estimated to be ~16.6 at.%. Such high-concentration H in the a-Si:H films was also regarded as being a favorable factor for crystallization of a-Si:H^15^,^37^. Furthermore, the a-Si:H nanoparticles revealed in the TEM image (see Fig. 3(a)) favor the crystallization of a-Si:H. An increased probability of crystallization for the case of nanostructured silicon was theoretically predicted by the group of Giulia Galli^29^: based on the molecular dynamics simulation, the authors revealed that a large-area surface can accommodate the volume expansion induced by the phase transformation (crystallization), resulting in a decrease in the Gibbs free energy for formation of crystalline nucleus in the vicinity of the system surface. Kramer *et al*.^38^ calculated the crystallization temperature of 3–5 nm sized silicon nanoparticles from the transient particle energy balance by means of the plasma heating model, and found decreasing crystallization temperature (in the range of 500–750 K) with decreasing particle size. Makoto *et al*.^39^ experimentally observed that the temperature threshold (*T*_*c*_) for crystallization decreases as the particle size decreases: *T*_*c*_ values of 10, 8, 6, and 4 nm particles were 1273, 1173, 1073, and 773 K, respectively. Obviously, the temperature *T*_*eff*_ = 779 K estimated in our experiment (for a-Si:H containing numerous ~3 nm particles) is enough to meet the crystallization requirement of a-Si:H at nanoscale.

**Figure 5:**
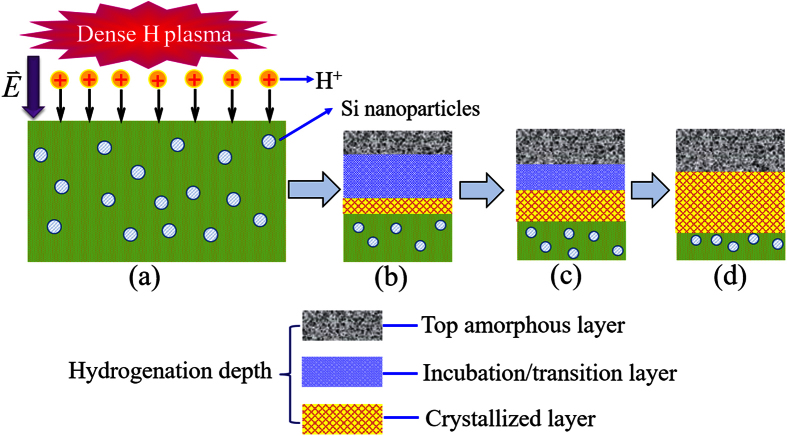
Schematic description of the plasma process, where the high-density hydrogen plasma-treated a-Si:H displays a time-dependent multilayered structure (Please refer to the text). The sheath electrical field and the nanoparticle structure facilitate the rapid crystallization process.

As a remark, this plasma treatment process is described schematically in Fig. 5. LFICP-generated high-density positive hydrogen ions accelerated by the sheath electric field 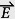 (~10^4^ V/cm) impinge on the treated surface and diffuse into the deep region of the films, as shown in Fig. 5(a). The filled circles represent Si nanoparticles embedded in the a-Si network. The treated a-Si:H displays a multilayered structure, including the top a-Si/SiO_x_ layer, the intermediate transition/crystallized layer, and the bottom a-Si:H layer (unreached layer). Figure 5(b–d) illustratively show a temporal evolution of the top a-Si/SiO_x_ layer, the intermediate incubation/transition layer, crystallized layer, and the bottom unreached layer. A short-time plasma treatment produced a pronounced top a-Si/SiO_x_, intermediate incubation/transition layer, and a thin crystallized layer. Extending the plasma treatment time leads to a reduced incubation/transition layer and a widened top a-Si/SiO_x_ layer, as shown in Fig. 5(c). The top a-Si/SiO_x_ layer ultimately approaches a nearly steady thickness with disappearance of the incubation/transition layer (see Fig. 5(d)). The crystallized layer increases linearly with the treatment duration. Such a simple process provides an effective way to achieve low-temperature and fast crystallization of a-Si:H, which could be another important crystallization method applied to crystallize amorphous materials after the electric-field-enhanced MIC technique^17^.

## Methods

The precursor a-Si:H films with thickness of ~2 μm were deposited on the double-side polished c-Si or glass substrate using the LFICP generator, whose main structure is schematically shown in the Supplementary Information. The power of RF generator (with frequency of 460 kHz, a set input power density of 17 mW/cm^3^ during deposition) is coupled into the deposition chamber sealed with a quartz window through a flat RF antenna connected with a matching/tuning network. The silicon and glass substrate were carefully cleaned by a standard chemical cleaning method and subsequently transferred into the deposition chamber through a load lock to avoid breaking the vacuum. The sample-holder is equipped with a thermocoax resistance, which allows monitoring and keeping the sample temperature at 100 °C during deposition. The base pressure of ~2 × 10^−4^ Pa in the deposition chamber was achieved through a combination of rotary and turbo-molecular pumps (TMP). The flux of silane and H_2_ gases was kept constant at 10 sccm (regulated by high-precision mass flow controllers (MFC)) with a constant flux ratio of [SiH_4_]/[H_2_]=1, and the gas temperature during discharging was monitored by another thermocouple located near the substrates. Some of the as-deposited a-Si:H samples were transferred into another clear LFICP chamber for the post-deposition hydrogen plasma treatment. The input RF power density and the sample-holder temperature for this stage are 42 mW/cm^3^, 300 °C, respectively. In order to investigate the time evolution of hydrogen in-diffusion in a-Si:H, the treatment duration was varied in the range of 0–30 minutes.

The thicknesses of the precursor a-Si:H thin films were measured directly from the cross-sectional scanning electron microscope (SEM) measurements by using a JEOL JSM-6700F field emission scanning electron microscope, and confirmed by the cross-sectional transmission electron microscopy (TEM) measurements. The TEM measurement was carried out through the use of a FEI Tecnai G2 20 transmission electron microscope equipped with LaB6 filament. In order to study the Si-Si bond configurations, micro-Raman scattering measurement was carried out with a Renishaw 1000 micro-Raman system fixed with a 514 nm Ar^+^ laser for excitation. Fourier transform infrared (FTIR) measurement was performed on a Perkin–Elmer FTIR 1725X spectrometer in the mid-infrared range from 400 to 4000 cm^−1^ with an increment of 1 cm^−1^ to explore the Si-H bonding configurations and extract hydrogen content in the films.

## Additional Information

**How to cite this article**: Zhou, H. P. *et al*. Hydrogen-plasma-induced Rapid, Low-Temperature Crystallization of µm-thick a-Si:H Films. *Sci. Rep.* **6**, 32716; doi: 10.1038/srep32716 (2016).

## Supplementary Material

Supplementary Information
