# Supplementary material for: Hydrogen-plasma-induced Rapid, Low-Temperature Crystallization of μm-thick a-Si:H Films
Source: Sci Rep. 2016 Sep 7;6:32716. doi: 10.1038/srep32716 (PMC5013535; doi:10.1038/srep32716)
Supplement: Supplementary Information [file srep32716-s1.pdf]

Supplementary Information

Hydrogen-plasma-induced Rapid, Low-Temperature Crystallization of µm-thick a-Si:H Films

H. P. Zhou, M. Xu*, S. Xu*, L. L. Liu, C. X. Liu, L. C. Kwek, and L. X. Xu

*Corresponding authors: hsuming_2001@aliyun.com (M. Xu), shuyan.xu@nie.edu.sg (S. Xu)

Schematic structure of the LFICP reactor:

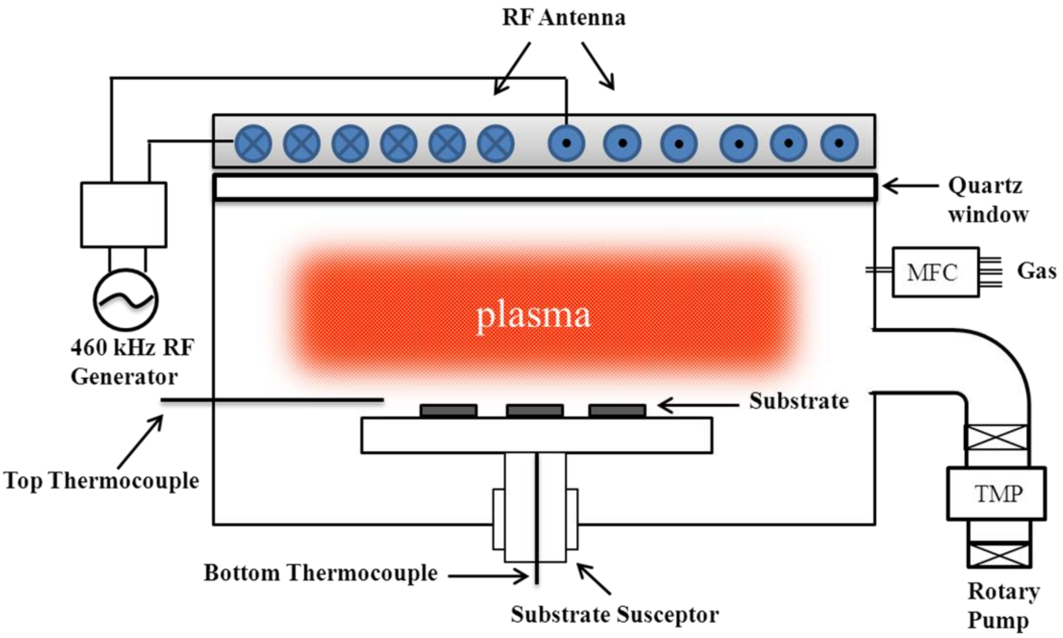

Figure S1. Schematic structure of the 460 kHz LFICP reactor. The top and bottom thermocouple are used to monitor the reactive gases and substrate temperatures, respectively.

The depth-dependent XPS measurements:

The X-ray photoelectron spectroscopy (XPS) measurements of the different layers in the 15-min plasma treated sample were conducted to elementally characterize the layers. As shown in Figure S2, layer (I) contains a substantial fraction of O and an insignificant fraction of C besides the primary element of Si.

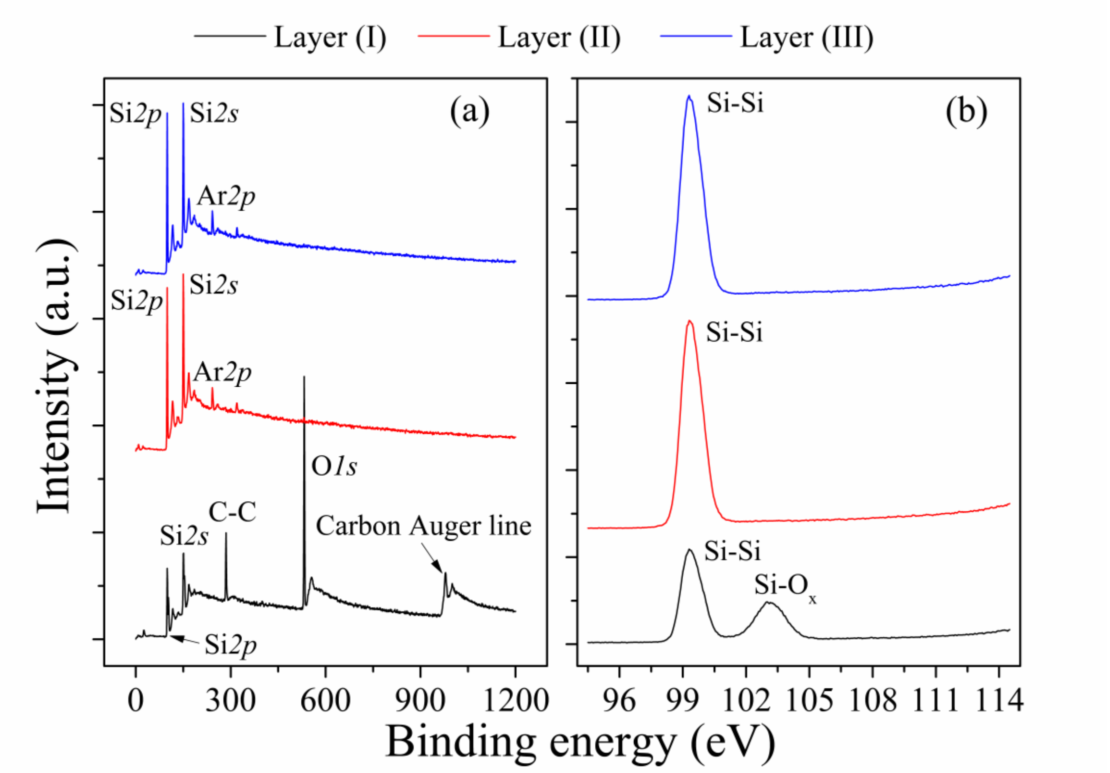

Figure S2. The survey scan XPS patterns of the different layers [see Figure 1(b)] of the 15-min plasma treated sample (a) and the narrow band scan of the state Si2p (b). The corresponding binding states are marked in the spectra.[s1]

Planar AFM images of the films:

The impinging hydrogen ions/atoms react with silicon with the occurrence of hydrogen insertion/etching on the surface and, hence the surface morphology changes due to hydrogen plasma exposure. Figure S4 shows the planar AFM images of a-Si:H treated for various durations and the calculated root-mean-square (RMS) roughness.

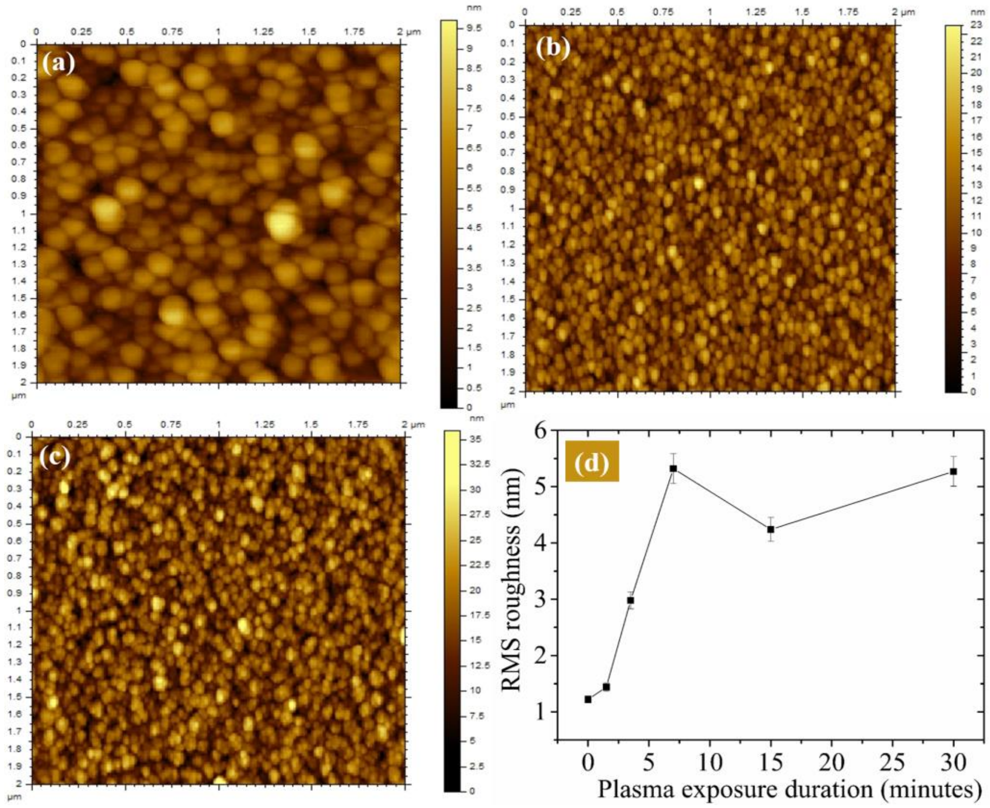

Figure S3. Surface AFM images taken in 2×2 μm^2 regions of the as-deposited film (a), 3.5-min treated (b), and 15-min treated (c) a-Si:H films. The RMS roughness values calculated from AFM measures are plotted against the plasma exposure duration in (d). Roughness increases pronouncedly from 1.2 nm to 5.3 nm within the treatment time of 0-7 minutes, and then fluctuates slightly after 7 minutes.

Calculation of the hydrogen content in a-Si:H films:

The bonded hydrogen concentration N_H in a-Si:H is estimated from the peak at ~ 640 cm^{-1} (Si-H wagging/rocking vibrational mode) using the following equation,[S2]

$$N_H = A_\omega \int \frac{\alpha(\omega)}{\omega} d\omega = A_\omega I_\omega, \qquad (1)$$

where $\alpha(\omega)$ is the absorption coefficient at frequency of ω, and the oscillator strength A_ω is 1.6×10^{19} cm^{-2}. The corresponding hydrogen content is defined as the ratio of the concentration of hydrogen atoms to that of hydrogen plus silicon atoms.

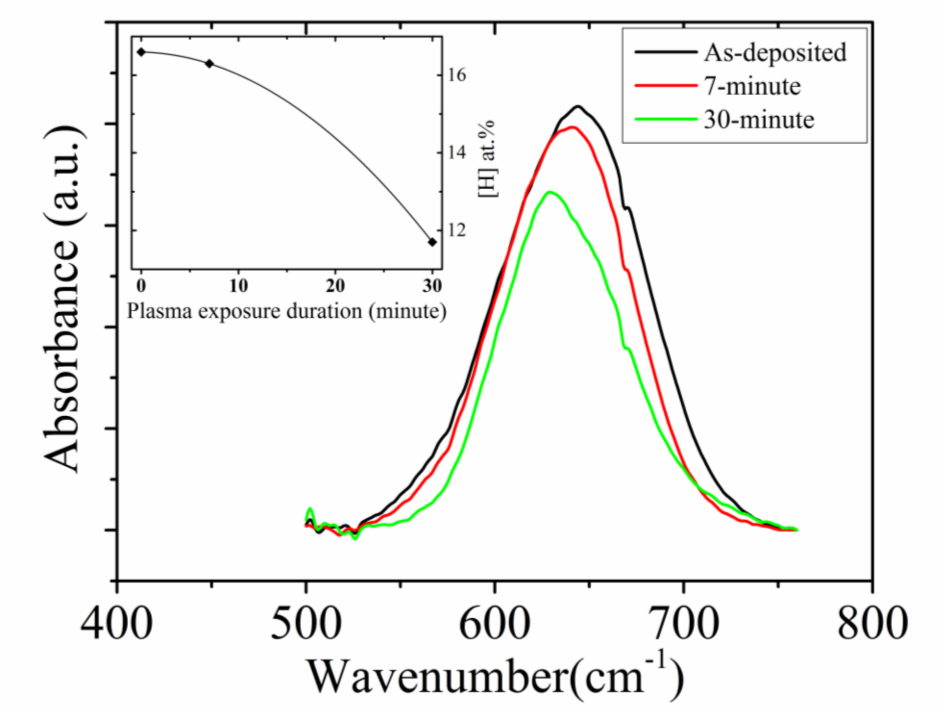

Figure S4. The wagging/rocking vibrational mode in the as-deposited and 7- and 30-min exposed films. The calculated hydrogen contents in the films are shown in the inset, where hydrogen content decreases with the plasma exposure duration. The line in inset is to guide the eyes.

References for Supplementary Materials

[s1] Moulder, J., Stickle, W., Sobol, P., and Bomben, K. Handbook of X-ray Photoelectron Spectroscopy (Physical Electronics, Minnesota, 1995).

[s2] Shanks, H., Fang, C. J., Ley, L., Cardona, M., Demond, F. J., and Kalbizer, S. Infrared Spectrum and Structure of Hydrogenated Amorphous Silicon. *Phys. Status Solidi* B **110**, 43-56, doi: 10.1002/pssb.2221000103 (1980).
